# Ethylene Response Factors Are Controlled by Multiple Harvesting Stresses in *Hevea brasiliensis*

**DOI:** 10.1371/journal.pone.0123618

**Published:** 2015-04-23

**Authors:** Riza-Arief Putranto, Cuifang Duan, Kuswanhadi, Tetty Chaidamsari, Maryannick Rio, Piyanuch Piyatrakul, Eva Herlinawati, Julien Pirrello, Florence Dessailly, Julie Leclercq, François Bonnot, Chaorong Tang, Songnian Hu, Pascal Montoro

**Affiliations:** 1 CIRAD, UMR DAP, Montpellier, France; 2 IBRIEC, Bogor, Indonesia; 3 RRI, CATAS, Danzhou, Hainan, China; 4 IRRI, Sembawa, Palembang, Indonesia; 5 Rubber Research Institute, Chatuchak, Bangkok, Thailand; 6 CIRAD, UMR BGPI, Montpellier, France; 7 BIG-CAS, Beijing, China; Institute of Crop Sciences, CHINA

## Abstract

Tolerance of recurrent mechanical wounding and exogenous ethylene is a feature of the rubber tree. Latex harvesting involves tapping of the tree bark and ethephon is applied to increase latex flow. Ethylene is an essential element in controlling latex production. The ethylene signalling pathway leads to the activation of Ethylene Response Factor (ERF) transcription factors. This family has been identified in *Hevea brasiliensis*. This study set out to understand the regulation of *ERF* genes during latex harvesting in relation to abiotic stress and hormonal treatments. Analyses of the relative transcript abundance were carried out for 35 *HbERF* genes in latex, in bark from mature trees and in leaves from juvenile plants under multiple abiotic stresses. Twenty-one *HbERF* genes were regulated by harvesting stress in laticifers, revealing an overrepresentation of genes in group IX. Transcripts of three *HbERF-IX* genes from *HbERF-IXc4*, *HbERF-IXc5* and *HbERF-IXc6* were dramatically accumulated by combining wounding, methyl jasmonate and ethylene treatments. When an ethylene inhibitor was used, the transcript accumulation for these three genes was halted, showing ethylene-dependent induction. Subcellular localization and transactivation experiments confirmed that several members of HbERF-IX are activator-type transcription factors. This study suggested that latex harvesting induces mechanisms developed for the response to abiotic stress. These mechanisms probably depend on various hormonal signalling pathways. Several members of HbERF-IX could be essential integrators of complex hormonal signalling pathways in *Hevea*.

## Introduction

Latex, a rubber-containing cytoplasm, is harvested by tapping soft bark tissues of *Hevea brasiliensis*. Latex production depends on the flow and regeneration of latex between two tappings. Ethephon, an ethylene releaser, is applied to the tapping panel to stimulate latex production. Ethephon application induces several biochemical changes in laticifers, such as sucrose loading [1], water uptake [2], and nitrogen assimilation or synthesis of defence proteins [3], involving a large number of ethylene-response genes [4]. Production of endogenous ethylene by tapping and exogenous ethylene by ethephon stimulation are likely to be sources of stress conducive to the biosynthesis of defence proteins and secondary metabolites, comprising rubber, in order to protect wounded laticifers [5]. Over a certain limit of environmental and harvesting stress, an intra-laticifer oxidative burst is generated. This oxidative stress leads to the peroxidation of membranes and the release of agglutinins such as Hevein from lutoids, which are defined as a polydispersed lysosomal vacuome [6]. Hevein is involved in the *in situ* coagulation of rubber particles [7]. This physiological syndrome, called tapping panel dryness (TPD), is responsible for substantial rubber production losses [8]. Ethylene therefore plays an ambivalent role that is conducive to latex production, but becomes unfavourable beyond a certain level.

The ethylene signal is perceived in plant cells through cascades of responses [9,10]. Ethylene Response Factors (ERFs) are the last known actor in the ethylene transduction pathway and regulate downstream ethylene-responsive genes. The ERF family belongs to the AP2/ERF superfamily. These transcription factor proteins have at least one AP2 conserved domain [11]. ERFs are trans-acting factors that bind to GCC or DRE/CRT *cis*-acting elements in the promoter region of target genes [12]. Many studies have shown that *ERF* genes are associated with plant development and responses to biotic and abiotic stress [13]. This family of genes has been extensively characterized, especially for responses to cold, dehydration, and low-oxygen sensing [14]. ERFs have been commonly classified as ethylene-dependent genes and regulate downstream target genes with or without interaction with other phytohormones [15]. The ERF family has been subdivided into ten groups with specific functions [16].

Recently, a comprehensive transcriptome led to the identification of 87 unique contigs related to *Hevea* ERFs, which were organized in 10 groups [17,18]. The characterization of these transcription factors has been carried out in different biological contexts, such as the somatic embryogenesis process [19], jasmonic acid-induced laticifer differentiation [20], and abiotic stress [21–23]. Although more recent characterization revealed a high accumulation of transcripts for some ERF groups (HbERF-II and HbERF-VII) in laticifers [18], their transcriptional regulation under abiotic stress related to the practice of latex harvesting have yet to be elucidated. Tapping can be considered as mechanical wounding and osmotic stress due to the loss of cytoplasm. Consequently, latex harvesting stress should involve several hormone signalling pathways such as ethylene, jasmonate, and ABA.

This paper set out to understand the regulation of *ERF* genes during latex harvesting in relation to abiotic stress and hormonal treatments. Analyses of the relative transcript abundance by real-time RT-PCR were carried out in latex, in bark from mature trees and in leaves from juvenile plants under multiple abiotic stresses. The Table 1 summarizes earlier work and current work on the 35 *Hevea brasiliensis ERF* genes tested in response to harvesting stress in bark and latex. A hierarchical clustering of gene expression profiles led to the identification of 21 *HbERF* genes regulated by ethylene (ET), methyl-jasmonate (MeJA) and dehydration in laticifers. Several members of *HbERF*-*IX* are induced by ethephon and/or tapping in latex and bark. Synergetic effects of ethylene and MeJA on transcript accumulation were observed for *HbERF-IXc4*, *HbERF-IXc5* and *HbERF-IXc6*. Pharmacological experiments using the inhibitor of ethylene action 1-methylcyclopropene (1-MCP) revealed that some ERFs are ethylene-independent. Subcellular localization and transactivation experiments suggested that several members of the HbERF-IX group are an activator type of transcription factors. This work is the first in-depth characterization of ERFs in a perennial tropical species and provides insight into the complex transcriptional regulations of ERFs. This study suggested that latex harvesting induces mechanisms developed for the response to abiotic stress. These mechanisms probably depend on various hormonal signalling pathways, such as ethylene and jasmonate, which greatly regulate *HbERF-IX* genes. Crosstalk between these complex hormonal regulations might play an important role in laticifers.

**Table 1:** Summary of 35 *Hevea brasiliensis ERF* genes tested in response to harvesting stress in bark and latex .

ERF group	Gene	Previous studies (Duan et al. 2013; Piyatrakul et al., 2014)					This work			
		Contig	Length (bp)	Gene expression marker in non-tapped trees	Phylogenetic analysis	Predicted miR-targeted ERFs	Number of introns	*Cis*-acting element-related hormonal pathways present in ERF promoters	Gene expression marker in tapped trees	
									Harvesting stress	Expression in latex
HbERF-I	*HbERF-Ib4*	CL1Contig10232	1983	Male flower			0	ET, JA, ABA, IAA, CK, GA		
	*HbERF-Ib7*	CL1Contig11231	1956				0	ET, JA, ABA, CK, GA	T, Eth	High
	*HbERF-Ib11*	CL13631Contig1	905	Male flower, bark, latex			0	ET, JA, ABA, IAA, CK, GA		
HbERF-II	*HbERF-IIb2*	CL17512Contig1	675	Embryo, leaf	ORA47		0	ET, JA, ABA, CK, GA	T, Eth	High
HbERF-III	*HbERF-IIIb1*	CL7809Contig2	1450	Latex			0	ET, JA, ABA, IAA, CK, GA	Eth	
	*HbERF-IIIe1*	CL1Contig2578	1434			miR894	0	JA, ABA, IAA, CK, GA		
HbERF-IV	*HbERF-IVa3*	CL10676Contig1	1324	Male flower, latex			0	ET, JA, ABA, CK, GA	T, Eth	High
HbERF-V	*HbERF-Va2*	CL1Contig20078	467				0	ET, JA, ABA, IAA, CK, GA		
HbERF-VI	*HbERF-VI1*	CL15015Contig1	844	Male flower			0	ET, JA, ABA, IAA, CK, GA	T	High
	*HbERF-VI3*	CL10440Contig2	1585	Embryo, leaf			0	ET, JA, ABA, IAA, CK, GA	T	High
	*HbERF-VI5*	CL1Contig16711	1616	Latex		miRn11		ET, JA, ABA, IAA, CK, GA	T, Eth	High
HbERF-VI-L	*HbERF-VI-L3*	CL932Contig9	1213	Male flower	CRF10		0	ET, JA, ABA, CK, GA	Eth	
	*HbERF-VI-L4*	CL5142Contig2	1918	Male flower			1	ET, JA, ABA, CK, GA	T	High
HbERF-VII	*HbERF-VIIa1*	CL1Contig8301	1014	Embryo, cotyledon			1	ET, JA, ABA, IAA, CK, GA		
	*HbERF-VIIa12*	CL1Contig1275	1825	Bark, latex	RAP2.12		2	ET, JA, ABA, IAA, CK, GA	T	High
	*HbERF-VIIa20*	CL1Contig726	1046	Leaf	RAP2.3	miR894	1	ET, JA, ABA, CK, GA	T	High
HbERF-VIII	*HbERF-VIIIa4*	CL1Contig10902	1126	Latex	ERF3		0	JA, ABA, CK, GA	T	
	*HbERF-VIIIa8*	CL1273Contig1	1009	Latex			0	ET, JA, ABA, CK, GA	T	
	*HbERF-VIIIa9*	CL1273Contig2	1270				0	ET, JA, ABA, CK, GA	T	High
	*HbERF-VIIIa10*	CL1257Contig1	1017		ERF11		0	ET, JA, ABA, IAA, CK, GA	T, Eth	High
	*HbERF-VIIIa12*	CL1Contig591	1106				0	ET, JA, ABA, CK, GA	T	High
	*HbERF-VIIIa13*	CL4149Contig1	945		ERF12		0	ET, JA, ABA, IAA, CK, GA	T, Eth	High
	*HbERF-VIIIa14*	CL4149Contig2	743				0	ET, JA, ABA, IAA, CK, GA	T, Eth	High
	*HbERF-VIIIb1*	CL1Contig9348	709		DRNL		0	ET, JA, ABA, IAA, CK, GA, OX	T	
HbERF-IX	*HbERF-IXa3*	CL8461Contig3	695	Latex	AtERF1		0	ET, JA, ABA, CK, GA	T, Eth	High
	*HbERF-IXb1*	CL6925Contig1	1158	Latex		miR408	0	ET, JA, ABA, IAA, CK, GA	T, Eth	High
	*HbERF-IXb2*	CL10861Contig1	736				0	ET, JA, ABA, CK, GA		
	*HbERF-IXb3*	CL1054Contig1	1221	Latex	ERF5/MACD1		0	ET, JA, ABA, IAA, CK, GA	T	
	*HbERF-IXc1*	CL108Contig2	887				0	ET, JA, ABA, CK, GA	T	High
	*HbERF-IXc4*	CL4147Contig3	888	Bark	ERF1		0	ET, JA, ABA, IAA, CK, GA, OX	T, Eth	High
	*HbERF-IXc5*	CL4147Contig1	934	Bark	ERF1	miR894	0	ET, JA, ABA, CK, GA, OX	T, Eth	High
	*HbERF-IXc6*	CL6690Contig1	1059		ORA59	miR1511	0	ET, JA, ABA, CK, GA	T, Eth	
HbERF-X	*HbERF-Xa2*	CL98Contig4	1526	Immature female flower	RAP2.6-LIKE		1	ET, JA, ABA, IAA, CK, GA	T	
	*HbERF-Xa8*	CL17010Contig1	737		ABR1	miRn11	1	ET, JA, ABA, CK, GA	T, Eth	High
	*HbERF-Xb1*	CL6880Contig1	1097		RRTF1		0	ET, JA, ABA, IAA, CK, GA	T, Eth	High

The table summarizes earlier works by Duan et al., 2013; Piyatrakul et al., 2014 and current work. T: tapping, Eth: ethephon. ET: ethylene, JA: jasmonic acid, ABA: abscisic acid, IAA: indole acetic acid, CK: cytokinin, GA: gibberellic acid, OX: oxidative stress.

## Materials and Methods

### Plant material

Latex and bark tissues were collected after one year of tapping from 8-year-old mature rubber trees of clone PB 260 at the Sembawa Station of the Indonesian Rubber Research Institute, P.O Box 1127, Palembang 30001, Indonesia (Latitude -2.9279748; Longitude 104.55701850000003) A half-spiral cut was made downward on the trees to release latex. Ethephon (Eth) was applied to the bark once a month at a concentration of 2.5%. Samples were collected 24 hours after ethephon treatment.

*In vitro* plantlets of clone PB 260 were obtained by somatic embryogenesis with line CI07060 using the method developed by CIRAD (Latitude 43.610769, Longitude 3.8767159999999876) [24]. The plantlets were acclimatized and grown for 3 months in a greenhouse at a temperature of 27°C with 45% humidity. Several treatments mimicking abiotic stress were carried out, such as wounding (W), MeJA, ET, dehydration and cold at 1, 4, 8 and 24 hours. The leaves were mechanically wounded by squeezing their entire surface with pincers, whilst the bark was wounded every 0.5 cm by scarification with a razor blade. The treatment with ET and MeJA was carried out by placing the plants in a 300 L open-door Plexiglas box overnight before treatment. Five parts per million of pure ET gas (1.5 mL/300 L) was injected into the sealed air-tight box. A concentration of 100 μL of liquid ≥95% MeJA solution was diluted in 500 μL of absolute ethanol and then placed on Whatman paper inside the box for gas release. Control plants used for the ET and MeJA treatments were placed in the box and exposed to air only. The dehydration treatment was carried out by taking the plants out of their pots and leaving them dry under laminar air flow. The cold treatment was carried out by placing the plants inside a cold chamber at 4°C. Each treatment was started at 7:00 am and leaf samples were collected after 1 h, 4 h, 8 h and 24 h of treatment.

Buds of the rubber clone PB 260 were grafted onto seedling rootstocks. The budded plants were grown at 28°C in a greenhouse under natural light. Three-month-old epicormic shoots from budded plants and leaves were treated at the same time. Leaves and bark of these plants were subjected or not to various factors, either alone or in combination (treatments): mechanical wounding, MeJA, ET, WxMeJA, WxET, MeJAxET, WxMeJAxET. Leaves were mechanically wounded by squeezing the entire surface of the leaves with pincers, whilst the bark was wounded every 0.5 cm by scarification with a razor blade. For the ET and MeJA gas treatments, plants were placed in a 300 L open-door Plexiglas box overnight before the treatment. One ppm of pure ethylene gas (0.3 mL/300 L) was injected into the sealed air-tight box. The concentration was controlled by gas chromatography (Type HP 5280 with FID detector). For the methyl jasmonate treatment, 20 μL of liquid ≥ 95% methyl jasmonate solution was diluted in 500 μL of absolute ethanol, and then placed on Whatman paper inside the box for gas release. Each treatment was compared to a specific control sampled at the same time and with the same culture conditions in three biological replications. Plants were treated at 8:00 am and tissues were collected 4 hours after treatment based on various preliminary kinetics experiments [4]. An inhibitor of ET action, 1-MCP, was used to demonstrate the specific effect of ET. Plants were pre-treated for 16 h with 1 ppm 1-MCP prepared with 480 mg of a 1-MCP-releasing powder dissolved in 7.2 mL of water. After ventilation, plants were then treated with 1 ppm of ET for 4 h. Control plants used for the ET, 1-MCP/ET treatment, were placed in the box and exposed to air only. In order to avoid variation due to the daytime and to biological development, each treatment was compared with a specific control sampled at the same time and with the same culture conditions in three biological replications. After treatment, bark tissues were collected and immediately frozen in liquid nitrogen and stored at -80°C until RNA extraction.

### Total RNA isolation

Total RNAs were isolated using the caesium chloride cushion method adapted from Sambrook [25] by Duan and coll. [4]. One gram of fresh matter was ground and transferred to a tube containing 30 mL of extraction buffer consisting of 4 M guanidium isothiocyanate, 1% sarcosine, 1% polyvinylpyrrolidone and 1% ß-mercapto-ethanol. After homogenization, tubes were kept on ice and then centrifuged at 10,000 g at 4°C for 30 minutes. The supernatant was transferred to a new tube containing 8 mL of 5.7 M CsCl. Ultracentrifugation in a swinging bucket was carried out at 89,705 g at 20°C for 20 hours. The supernatant and caesium cushion were discarded whilst the RNA pellet was washed with 70% ethanol. After 30 minutes of air drying, the pellet was dissolved in 200 μL of sterile water. Although DNA could not cross the caesium cushion for this centrifugation condition, DNA contamination was checked by PCR amplification using primers of the *Actin* gene including the intron sequence. RNAs were conserved at -80°C.

### Primer design and analysis of transcript abundance by real-time RT-PCR

Several rules were applied in order to reduce the risk of error in relative gene expression data. The integrity of total RNA was checked by electrophoresis. Primers were designed at the 3’ side of each sequence in order to reduce the risk of error due to short cDNA synthesis using the Primer 3 module of Geneious. Real-time PCR amplification and the fusion curve were carried out using a mix of cDNAs in order to check the specificity of each pair of primers. Sequencing of the PCR amplicon was carried out to verify the product sequence. Primer sequences are listed in S1 Table.

cDNAs were synthesized from 2 μg of total RNA to the final 20 μL reaction mixture using a RevertAidTM M-MuLV Reverse Transcriptase (RT) kit according to the manufacturer's instructions. Full-length cDNA synthesis was checked on each cDNA sample by PCR amplification of the Actin cDNA using primers at the cDNA ends. Quantitative gene expression analysis was finally carried out by real-time RT-PCR using a Light Cycler 480. Real-time PCR reaction mixtures consisted of 2 μL RT product cDNA, 0.6 μL of 5 μM of each primer, and 3 μL 2×SYBR green PCR master mix in a 6-μL volume. PCR cycling conditions comprised one denaturation cycle at 95°C for 5 min, followed by 45 amplification cycles (95°C for 20 s, 60°C for 15s, and 72°C for 20s). Expression analysis was performed in a 384-well plate. Samples were loaded using an automation workstation.

Real-time PCR was carried out for eleven housekeeping genes in order to select the most stable gene as the internal control for all samples (*HbelF1Aa*, *HbUBC4*, *HbUBC2b*, *HbYLS8*, *HbRH2b*, *HbRH8*, *HbUBC2a*, *HbalphaTub*, *Hb40S*, *HbUbi*, *HbActin*) (S2 Table). *HbRH2b* was selected as the best reference gene according to its stability in tissues from various treatments in mature trees and juvenile trees. The homogeneity of the *HbRH2b* gene Cp confirmed that it could be used as an internal reference gene. The *HbRH2b* gene was amplified in each reaction plate in parallel with target genes. The transcript abundance level for each gene was relatively quantified by normalization with the transcript abundance of the reference *HbRH2b* gene. Relative transcript abundance took into account primer efficiencies. All the normalized ratios corresponding to transcript accumulation were calculated automatically by Light Cycler Software version 1.5.0 provided by the manufacturer using the following calculation: Normalized Ratio = Efficiency ^-Δ*(Cp target-Cp RH2b)*^.

### Statistical analysis for the comparison of relative transcript abundance and for the analysis of interactions in mature trees and in juvenile budded plants

Each relative transcript abundance value was the mean of three biological replicates. Statistical analysis was performed after logarithmic transformation of raw data. The comparison of relative transcript abundances between treated and control plants was carried out using an ANOVA followed by a Fisher test.

The experimental design for mature trees included three factors (tapping, ethephon and a combination of both). The analysis of interactions between treatments was performed by an ANOVA followed by a Newman-Keuls test. The level of interaction between tapping (T), ethephon (Eth) and a combination of both (TxEth) was assessed for each tested gene in a variance table.

The experimental design for juvenile budded plants included three factors (wounding, methyl jasmonate, and ethylene) alone and in combination leading to eight treatments (Control (C), W, MeJA, ET, WxMeJA, WxET, MeJAxET, WxMeJAxET). The experimental unit was one plant. The level of expression was calculated as the ratio between the mean values of relative transcript abundances of treated and control plants. It was considered as an up-regulation when the ratio was >1.0, and a down-regulation when the ratio was <1.0. The ratio with a p-value of ≤ 0.05 was adopted as significant for down- or up-regulation. The level of interaction between W, MeJA and ET was assessed for each tested gene in a variance table.

Both analyses on mature trees and juvenile budded plants generated variance tables that included F values for each interaction and the corresponding P-values were noted as follows: <0.001 (***); <0.01 (**); <0.05 (*); <0.1 (°).

### Construction of hierarchical gene regulation profiles between harvesting stress in mature trees and abiotic stress in juvenile plants

The profiles of gene regulation between harvesting stress in mature trees and abiotic stress in juvenile plants was combined and hierarchized, based on gene induction related to tapping and ethephon in latex and bark. The level of expression of genes in latex was filtered from high to low, and other data from bark of mature plants and leaves of juvenile plants were automatically hierarchized. The level of expression was calculated as the ratio between the mean values of relative transcript abundances of treated and control plants, tapped and non-tapped plants, ethephon and non-ethephon-stimulated plants. It was considered as an up-regulation when the ratio was ≥ 5.0, and a down-regulation when the ratio was ≤ 0.2. For juvenile plants, data were selected from kinetic expression by considering the tendency of gene expression in response to stress. Data were aligned and modified into a standard colour code based on their up-regulation or down-regulation expression. No statistical analysis was performed on juvenile plants. For mature trees, the previous analysis of interactions (T, ET, TxET) was employed to validate significant data for the tapping or ethephon effect for each gene.

### Genomic scaffold analysis and in silico search for putative regulatory elements

The presence of introns was checked in the coding sequence of genomic scaffolds related to *HbERFs* by using the sequence-to-sequence alignment module of Geneious. The *Hevea* genomic scaffold was provided for the *HbERFs* by the CATAS-BIG *Hevea* Genome Project. The *Hevea* genome from clone CATAS 7-33-97 was assembled in 7787 scaffolds with a N50 size of 1,281,786 bp. *In silico* promoter analysis for six genes of HbERF-IX was conducted with the online tool PLACE: http://www.dna.affrc.go.jp/PLACE/signalscan.html [26]. A 2000 bp sequence upstream from the start codon was scanned for the presence of putative *cis*-acting regulatory elements using the database associated search tools. The number of copies for each *cis*-acting element was then counted.

### Subcellular localization and transcriptional activity tests by transient expression in a single cell system

Tobacco protoplasts were used in the subcellular localization because *Hevea* protoplasts have short viability. GFP N-terminal fusions were obtained with *HbERF-VIIIa13* and *HbERF-IXc6* and used for tobacco protoplast BY-2 transfection according to Chaabouni and coll. [27]. The subcellular location of the fluorescence was determined after 20 hours using confocal microscopy.

A transactivation experiment was carried out according to the procedure published by both Chaabouni and Pirrello [27,28]. A synthetic reporter construct (4XGCC-GFP) was used [28]. Effector constructs were generated by fusing the 35S promoter to the CDS of the genes (*HbERF-IXc4*, *HbERF-IXc5* and *HbERF-IXc6*). For transient assays, tobacco (Nicotiana tabacum) BY-2 protoplasts were co-transformed with reporter and effector constructs [27]. Transformation assays were performed in three independent replicates. After 16 h, GFP expression was analysed and quantified by flow cytometry. Data were analysed using Flowing software. For each sample, 100–400 protoplasts were gated on forward light scatter (FSC) and side light scatter (SSC) to check the size and the structure of protoplasts and eliminate debris from the analysis. The GFP fluorescent protoplasts used for the calculation of GFP activity were selected within a gate. This gate was previously defined by the comparison between transformed protoplasts (co-transformation with the effector plasmid lacking the HbERF coding sequence) and non-transformed protoplasts. The green fluorescence detected in the defined gate showed a marked hook. The GFP fluorescence per population of cells corresponded to the average fluorescence intensity of the cell population after subtraction of autofluorescence determined with non-transformed BY-2 protoplasts. The data were normalized using an experiment with protoplasts transformed with the reporter vector in combination with the vector used as the effector plasmid, but lacking the HbERF coding sequence. The ratio of GFP activities between GCC::GFP and mGCC::GFP constructs revealed the capacity of transcription factors to activate (ratio > 1) or repress (ratio < 1) the GCC promoter.

## Results

### Analysis of relative transcript abundance of the *Hevea AP2/ERF* genes during latex harvesting

**Fig 1:**
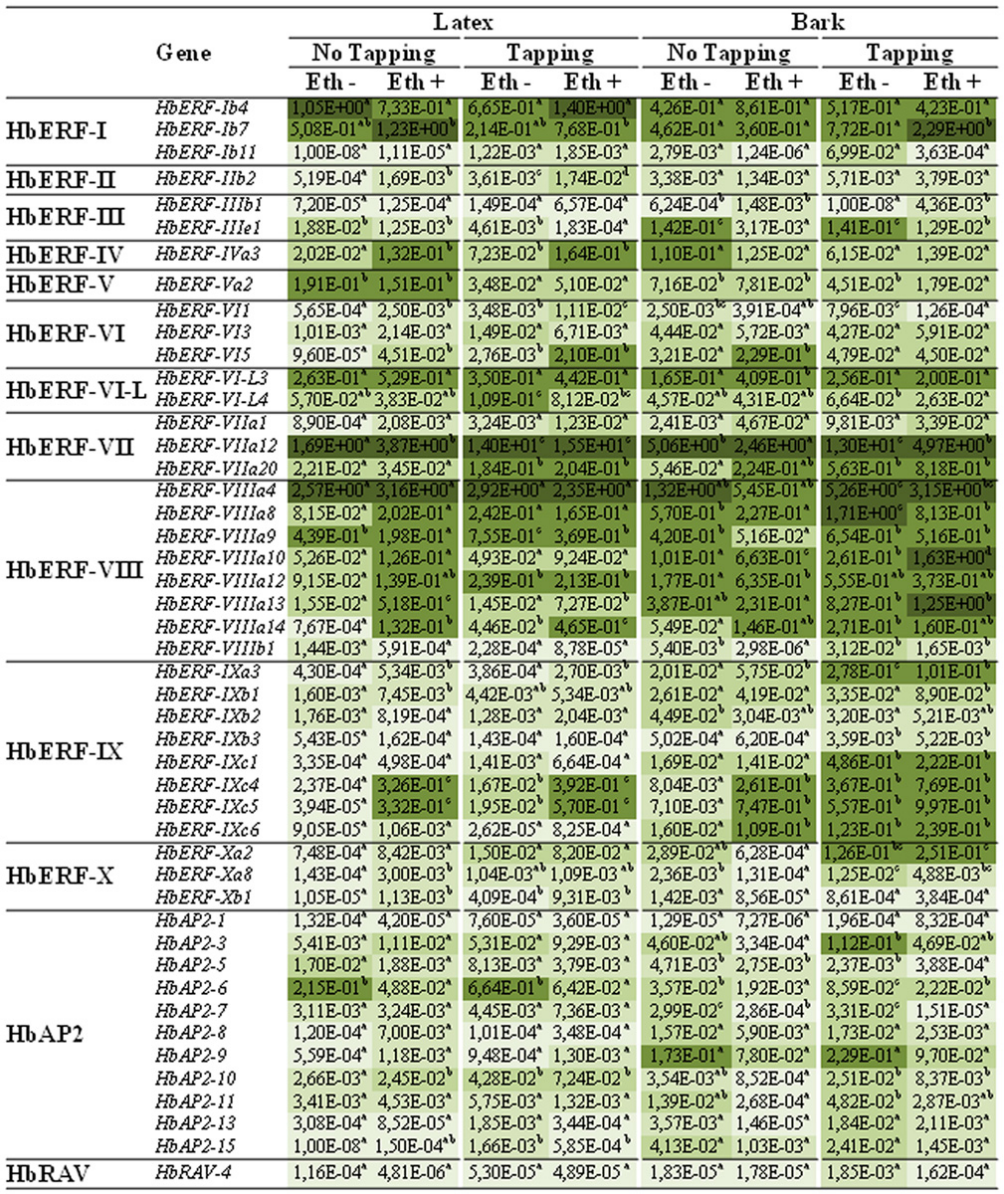
Relative transcript abundance profile of 47 *AP2/ERF* genes of *Hevea brasiliensis* in latex and bark tissues of mature trees during latex harvesting. Tapping without ethephon (Eth-) and with ethephon (Eth +) were applied in both studied tissues. Heat map representation was used for values ranging as follows ≥ 1, 10–1, 10–2, 10–3 and ≤ 10–4 from dark to light green.

Specific amplification of 47 of the 114 *Hevea* genes from the *AP2/ERF* superfamily including *AP2*, *ERF* and *RAV* genes was validated by sequencing [18].The relative transcript abundance for these 47 genes was analysed in latex and bark of 1-year-old tapped trees treated or not by ethephon (Fig 1). Thirty-seven genes were differentially regulated according to the ANOVA analysis (S3 Table). Of all the studied genes, *HbRAV-4* had a low relative transcript abundance under all conditions with a value lower than 10^-2^, equally for the genes of the AP2 family, except for *HbAP2-3*, *HbAP2-6*, and *HbAP2-9*. Seven out of eight genes for ERF group VIII (*HbERF-VIIIa4*, *HbERF-VIIIa8*, *HbERF-VIIIa9*, *HbERF-VIIIa10*, *HbERF-VIIIa12*, *HbERF-VIIIa13*, and *HbERF-VIIIa14*) had a high relative transcript abundance in all tissues and treatments with a value higher than 10^-1^, whereas five out of eight group IX genes (*HbERF-IXa3*, *HbERF-IXc1*, *HbERF-IXc4*, *HbERF-IXc5*, and *HbERF-IXc6*) had a high transcript abundance specifically due to tapping in bark. More particularly, the *HbERF-VIIa12* gene had a high relative transcript abundance in latex and bark, regardless of the treatments.

### Effect of tapping, ethephon and a combination of tapping and ethephon on the relative transcript abundance of *Hevea AP2/ERF* genes

**Fig 2:**
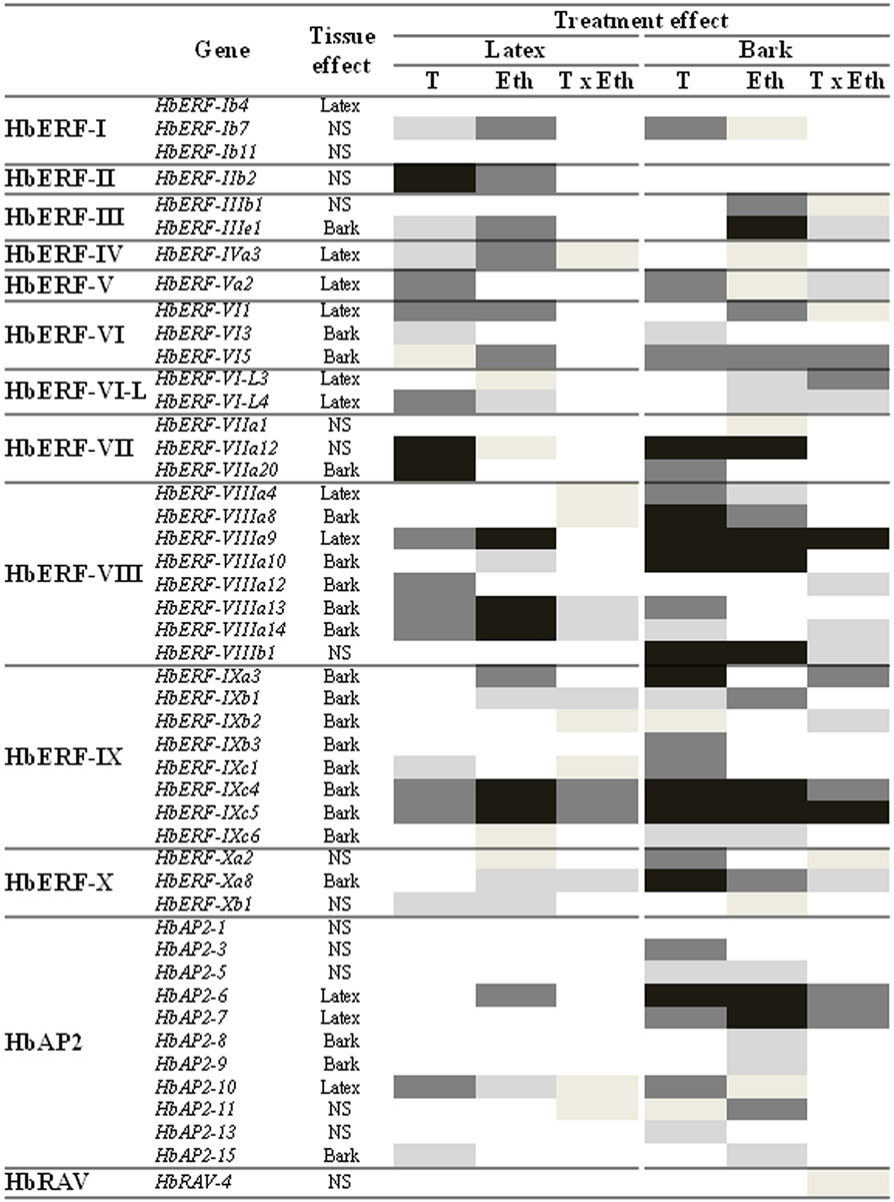
Interaction analysis profile of 47 *AP2/ERF* genes of *Hevea brasiliensis* in latex and bark tissues of mature trees during latex harvesting. P-values are indicated from black to white as follows: 0.001; 0.01; 0.05; 0.1 and non-significant data. T: tapping; Eth: ethephon; TxEth: combination of both.

The effects of tapping, ethephon and T x Eth interaction on the relative transcript abundance of *Hevea AP2/ERF* genes in latex and bark were studied by analysing the variance table of 47 genes (Fig 2, S4 Table). The tissue effect was significant for 32 of the 47 genes. Eleven genes had a higher relative transcript abundance in latex, as opposed to 21 genes in bark. Notably, all eight genes of ERF group IX were significantly changed in bark. In general, the effects of treatments were found to be stronger in bark. Thirty genes, as opposed to 21, showed a stronger effect of tapping in bark compared with latex. Seven genes (*HbERF-IIb2*, *HbERF-IIIe1*, *HbERF-IVa3*, *HbERF-VI1*, *HbERF-VI-L4*, *HbERF-VIIIa12*, and *HbAP2-15*) were induced specifically in latex. Thirty genes, as opposed to 24, had a stronger ethephon effect in bark compared with latex. Lastly, the combination effect was greater in bark compared with latex: 20 genes as opposed to 13, respectively.

### Fold change in the transcript abundance of *Hevea AP2/ERF* genes in response to abiotic stress in juvenile plants

**Fig 3:**
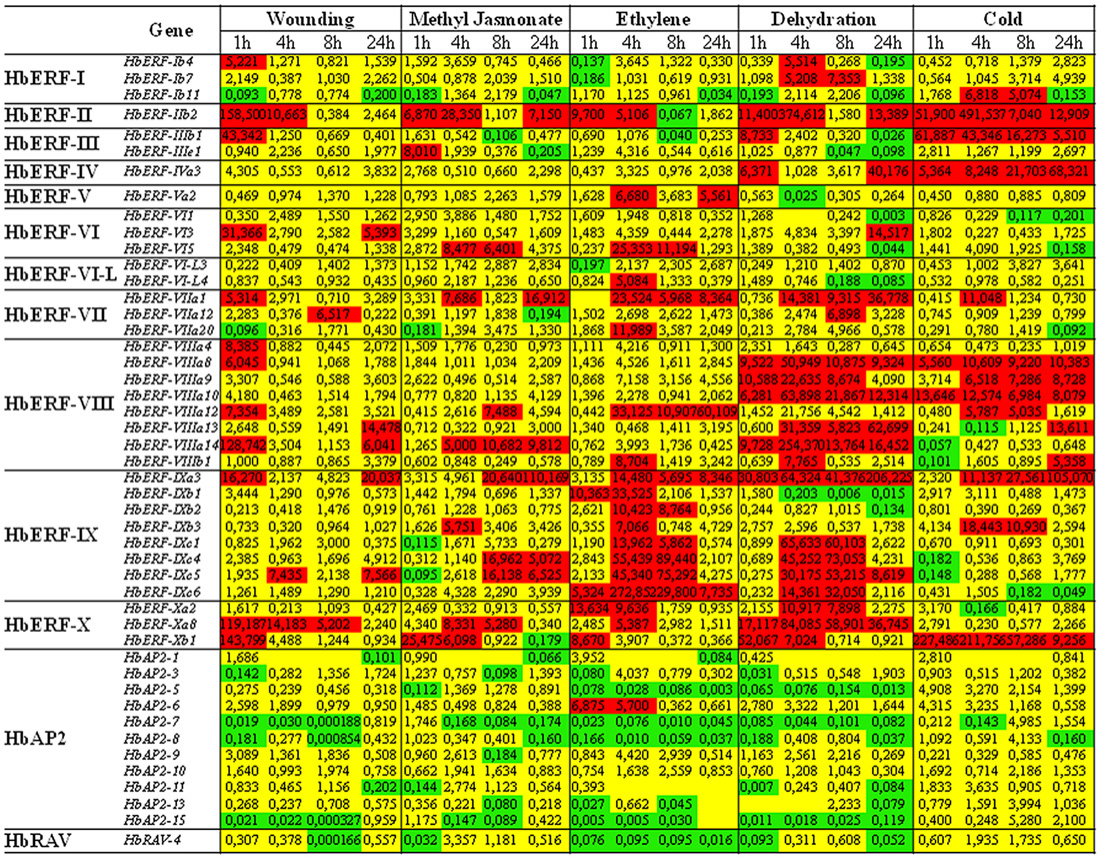
Gene regulation profile of 47 *AP2/ERF* genes of *Hevea brasiliensis* under various types of abiotic stress in leaves of juvenile plants. Up-regulated genes are shown in red with a threshold value ≥ 5; down-regulated genes are shown in green with a threshold value ≤ 0.2. The non-significant genes are shown in yellow.

Juvenile plants were a simple system for studying independently the effect of abiotic stress and hormone treatments on the relative transcript abundance. Leaves were chosen because of the sensitivity of that tissue. The ratio between the relative transcript abundances of treated and control plants was monitored 1, 4, 8 and 24 hours after treatments for 47 genes in leaf tissues of 7-month-old plants grown under conditions of various types of abiotic stress (Fig 3). A fold change threshold of 5 was selected to identify induced genes (ratio >5 in red) and down-regulated genes (ratio <0.2 in green). All *HbERF* genes were induced by at least one treatment, whereas the *HbAP2* and *HbRAV* genes showed a low ratio of transcript abundance in response to any stress, except for *HbAP2-6* in response to ethylene. Several ERF genes differentially responded to stress. High gene induction was noted for two ERF groups, in response to ethylene and dehydration for ERF group IX, and in response to dehydration and cold for ERF group VIII, respectively.

### Hierarchized transcript abundance ratios of *Hevea AP2/ERF* genes in response to harvesting and abiotic stress

**Fig 4:**
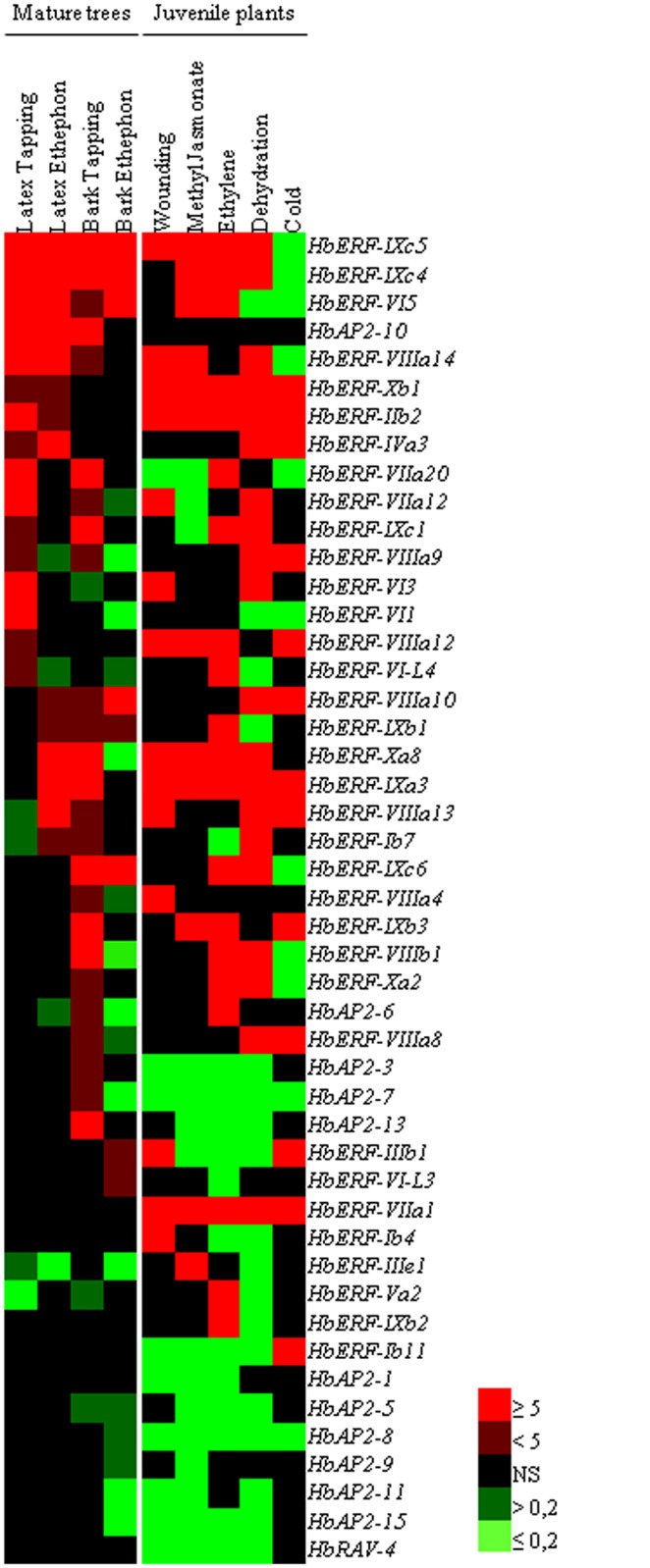
Ratios of gene expression for 47 *AP2/ERF* genes of *Hevea brasiliensis* in response to latex harvesting stress in mature trees and various types of abiotic stress in juvenile plants. Up-regulated genes are shown in bright red for a threshold value ≥ 5, dark red for a value 2<n<5, and down-regulated genes are shown in bright green for a threshold value ≤ 0.2, dark green for a value 0.5>n>0.2. The non-significant fold changes are shown in a dark colour.

The ratios of relative transcript abundance values for mature trees and juvenile plants in response to harvesting and abiotic stress were hierarchized from high to low ratios in latex in order to highlight genes that were highly regulated in that tissue (Fig 4). Three clusters of genes were highlighted. The first cluster consisted of thirty-four genes having a high ratio in response to harvesting. Most of the genes induced by harvesting stress were also induced by one or more types of abiotic stress, except *HbAP2-3*, *HbAP2-7*, *HbAP2-10*, *HbAP2-13*, *ERF-VI1* and *HbERF-VIL3*. The second cluster contained six genes (*HbERF-Ib4*, *HbERF-Ib11*, *HbERF-IIIe2*, *HbERF-Va2*, *HbERF-VIIa1* and *HbERF-Xb2*) that specifically responded to abiotic stress in the leaves of juvenile plants. The last cluster contained seven genes (6 *HbAP2* and 1 *HbRAV* genes) with a drop in transcript abundance in response to most of the abiotic stresses in leaves.

For the first cluster, twenty-one *HbERF* genes and one *HbAP2-10* gene were induced by harvesting stress in latex. A majority of the 16 *AP2/ERF* genes induced by tapping in latex was also induced by abiotic stress in leaves (7 by wounding, 7 by MeJA, 12 by ET, and 10 by dehydration). Of the 14 *AP2/ERF* genes induced by ethephon in latex, 8 were also induced by ET in leaves. Conversely, 5 ET-responsive genes in leaves were not induced by ethephon in latex. The *HbERF* genes from group IX and group VIII were the largest represented groups with 5 genes of each group induced by harvesting stress in latex and abiotic stress. Transcripts of *HbERF-IXc4* and *HbERF-IXc5* genes were the most dramatically accumulated in response to stress.

### Gene structure and *in silico* promoter analysis of *HbERF-IX* genes

To understand the gene structure of *HbERF* genes, transcript sequences were compared and aligned with *Hevea* genomic scaffolds provided by BIG and CATAS. Thirty-three scaffolds corresponding to 35 contigs were annotated. This analysis revealed a structure of genes with one or two exons (S5 Table). All analysed sequences from *ERF-IX* genes showed a uniform structure with 1 exon. For other groups, *HbERF* genes had no strict gene structure with 1 or 2 exons. For instance, *HbERF-VI-L4*, *HbERF-VIIa1*, *HbERF-VIIa12*, *HbERF-VIIa20*, *HbERF-Xa2* and *HbERF-Xa8* had 2 exons.

The *in silico* analysis of the 2000 bp region upstream of the start codon (ATG) of 35 *HbERF* genes was carried out using PLACE (S6 Table). Forty-five putative *cis-*acting regulatory elements were identified on these promoter sequences. The work was then focused on 8 *HbERF-IX* genes, which corresponded to 7 genomic scaffolds. The number of copies of *cis-*acting elements could reach 59 for the 7 analysed scaffolds. A large number of *cis-*acting regulatory elements were associated with hormone signalling pathways, metabolic activities and plant development. Sixteen of the 45 *cis-*acting elements were related to hormone and stress response: ERE, GCC-box, DRE/CRT I, DRE/CRT II, LTRE, JERE, E-box, W-box, ABRE, erd1, ARF, ARR1, GARE, Pyrimidine box, MYB, ARE1 (Table 2). All the members of HbERF-IX were predicted to have a primary ethylene response element (ERE). All members also had at least one GCC, DRE/CRT or LTRE box except *HbERF-IXc6*, revealing putative regulation by another ERF. These promoters had a large number of *cis*-acting elements involved in jasmonate (up to 47 elements), cytokinin (up to 43 elements) and gibberellin (up to 22 elements) signalling pathways. Finally, only *HbERF-IXc4* and *HbERF-IXc5* promoters harboured *cis-*acting elements for oxidative stress such as the Antioxidant Response Element 1 (ARE1).

**Table 2:** Number of copies of *cis*-acting regulatory elements involved in hormonal signalling in the HbERF-IX promoter region using an *in silico* pattern matching search against PLACE.

Plant hormones	Regulatory element	Core motif	Gene and corresponding scaffolds							
			*HbERF-IXa3*	*HbERF-IXb1*	*HbERF-IXb2*	*HbERF-IXb3*	*HbERF-IXc1*	*HbERF-IXc4*	*HbERF-IXc5*	*HbERF-IXc6*
			sc139	scaff2511	scaff2511_2	sc17055	sc424	sc16383	sc106	scaff8135
Ethylene	ERE	AWTTCAAA	1	1	1	1	2	1	4	3
	GCC-box	GCCGCC	1	1	1	0	0	1	2	0
	DRE/CRT I	RYCGAC	0	2	2	0	1	0	0	0
	DRE/CRT II	RCCGAC	0	0	3	0	2	0	0	0
	LTRE	CCGAC	0	0	7	1	2	0	1	0
Jasmonate	JERE	CGAC	2	3	1	4	7	2	2	0
	MYC (E-box)	CANNTG	22	20	20	18	20	24	24	18
	W-box	TGAC	47	14	23	23	33	18	31	12
Abscisic acid	ABRE	ACGTG	15	1	1	5	6	8	0	0
	erd1	ACGT	16	2	2	4	8	4	8	4
Auxin	ARF	TGTCTC	0	2	0	1	0	1	0	0
Cytokinin	ARR1	NGATT	21	28	27	22	32	43	17	20
Gibberellins	GARE	TAACAAR	3	1	1	1	1	1	4	1
	Pyrimidine box	CCTTTT	7	3	2	2	6	2	2	4
	MYB	CNGTTR	18	30	5	17	22	10	19	14
Oxidative stress	ARE1	RGTGACNNNGC	0	0	0	0	0	1	1	0

The analysis was carried out in the -2kb region upstream of the start codon (ATG).

### Subcellular localization of HbERF-IXc4, HbERF-IXc5 and HbERF-IXc6 and transactivation of a GCC synthetic promoter

**Fig 5:**
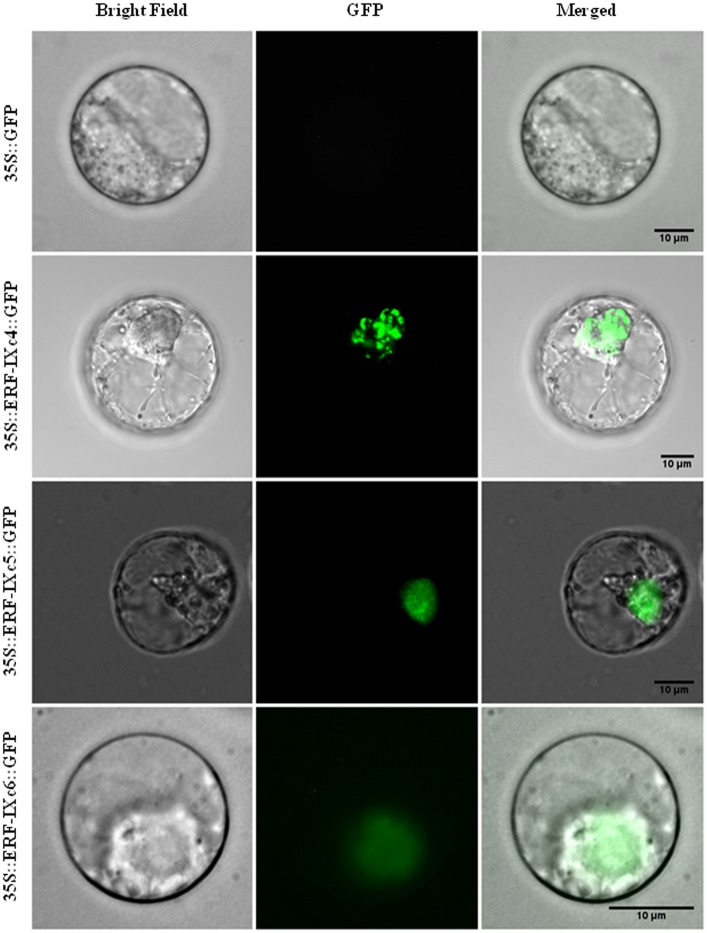
Subcellular localization of HbERF-IXs. The merged pictures of the green fluorescence channel (middle panels) and the corresponding bright field (left panels) are shown (right panels). Control cells expressing fluorescence absence are shown in the top panel. The scale bar indicates 10 μm.

**Fig 6:**
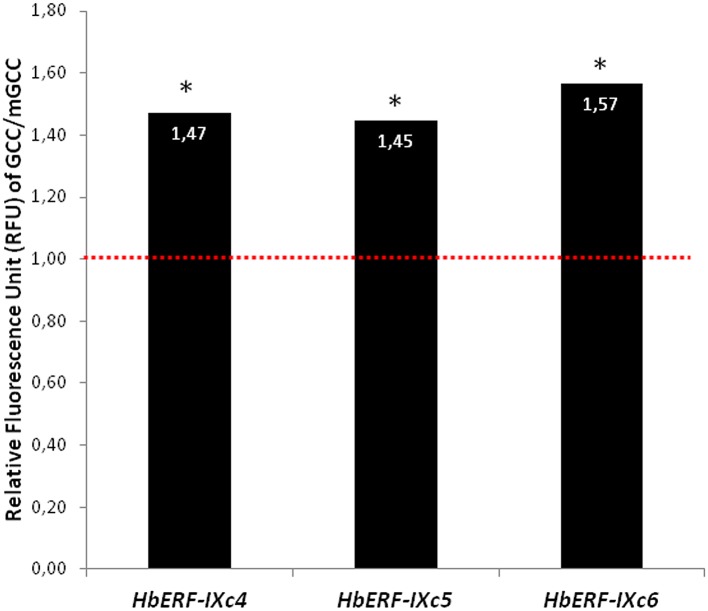
Transactivation of the synthetic GCC-box and mGCC containing promoters by HbERF-IXc4, HbERF-IXc5 and HbERF-IXc6 proteins. ERF candidates are activators (ratio > 1) or repressors (ratio < 1). (*) indicates a significant difference for the Student test (p<0.01).

In order to validate the function of HbERF-IXs as transcription factors, subcellular localization and transactivation experiments were carried out for three HbERFs (HbERF-IXc4, HbERF-IXc5, HbERF-IXc6). Transient expression of an HbERF/GFP translational fusion into BY-2 tobacco protoplasts revealed GFP activity of the fusion protein in the nucleus for each tested HbERF, in contrast with a pMDC83 empty control plasmid (Fig 5). In addition, the GFP reporter gene under the control of a synthetic promoter harbouring the GCC *cis*-acting element was transactivated by the three HbERF-IX candidates (Fig 6). All three HbERF-IXs showed a ratio greater than 1 (about 1.5) and could significantly be considered as activators.

### Synergistic effect of multiple stresses on *HbERF-IX* gene expression

The relative transcript abundance was analysed in bark tissue of juvenile plants for all *HbERF-IX* subgroup genes in response to mechanical wounding, MeJA and ET in order to check the specificity of gene induction in response to these factors. These factors were applied alone or in combination in order to study the interaction between ethylene and methyl jasmonate, and identify orthologues to ERF1 that dramatically respond to a combination of ET and MeJA. The expression ratio of 6 genes of the 8 tested (*HbERF-IXa3*, *HbERF-IXb1*, *HbERF-IXb2*, *HbERF-IXc4*, *HbERF-IXc5*, *HbERF-IXc6*) showed significant changes for at least one treatment compared with the control (Table 3). Transcripts of six *HbERF-IX* genes (*HbERF-IXa2*, *HbERF-IXb1*, *HbERF-IXb2*, *HbERF-IXc4*, *HbERF-IXc5*, *HbERF-IXc6*) were significantly accumulated in response to the combined W x MeJA x ET treatment. Interestingly, the *HbERF-IXc4*, *HbERF-IXc5* and *HbERF-IXc6* genes were induced by all the treatments with a ratio ranging from 2.95 to 3143. This expression ratio was dramatically increased when ET was combined with W and/or MeJA treatments. In particular for *HbERF-IXc4*, the ratio increased up to 39, 54 and 306-fold for the W, MeJA and ET treatments, respectively, and when ethylene was combined with MeJA this ratio jumped to 1754 and 3143. *HbERF-IXc5* and *HbERF-IXc6* showed similar but lower gene induction. By contrast, *HbERF-IXb2* was significantly down-regulated by these treatments. This gene was induced by ET, and conversely down-regulated (2–3 times) by W x MeJA and W x ET combinations.

**Table 3:** Analysis of the relative transcript accumulation of 8 *ERF* genes of *Hevea brasiliensis* from group IX by real-time RT-PCR in the bark of the control (C) and plants subjected to seven different treatments: (W) wounding; (MeJA) methyl jasmonate; (ET) ethylene, either individually or in a combination of treatments (T).

Gene	Treatments													
	W		MeJA		ET		W x MeJA		W x ET		MeJA x ET		W x MeJA x ET	
	ratio	*p-value*	ratio	*p-value*	ratio	*p-value*	ratio	*p-value*	ratio	*p-value*	ratio	*p-value*	ratio	*p-value*
	T/C		T/C		T/C		T/C		T/C		T/C		T/C	
*HbERF-IXa3*	2.60	0.13	1.63	0.20	0.65	0.37	2.35	0.14	9.88	0.06	7.68	0.06	124.96	0.05
*HbERF-IXb1*	0.40	0.14	2.53	0.37	8.84	0.01	0.41	0.14	0.32	0.11	4.09	0.03	30.35	0.01
*HbERF-IXb2*	0.73	0.27	0.62	0.24	5.26	0.01	0.36	0.02	0.42	0.03	1.95	0.14	12.40	0.05
*HbERF-IXb3*	0.06	0.16	0.69	0.80	0.21	0.57	0.28	0.63	0.08	0.22	1.18	0.78	0.14	0.37
*HbERF-IXc1*	0.35	0.49	2.85	0.64	0.15	0.89	1.08	0.25	3.03	0.16	0.93	0.33	32.76	0.25
*HbERF-IXc4*	38.81	0.01	53.90	0.00	306.20	0.003	38.82	0.01	396.07	0.001	1754.05	0.000	3143.14	0.003
*HbERF-IXc5*	5.38	0.03	2.95	0.04	38.53	0.002	4.36	0.02	224.85	0.001	120.67	0.004	502.54	0.02
*HbERF-IXc6*	17.09	0.01	29.52	0.01	57.90	0.003	4.28	0.08	124.79	0.002	257.63	0.002	588.72	0.01

It was considered as an up-regulation when the ratio was >1.0, and a down-regulation when the ratio was <1.0.

Interactions between wounding, ethylene and methyl jasmonate were studied through the variance in transcript abundance of the 8 *HbERF-IX* genes in response to the eight different combinations of factors. The expression of 6 genes was significantly changed by the W, MeJA and ET treatments, respectively (S7 Table). The relative transcript abundance of the *HbERF-IXa3*, *HbERF-IXb1* and *HbERF-IXc4* genes was significantly modified for all the three factors applied alone. *HbERF-IXb1* and *HbERF-IXb2* showed a significant effect of interactions between the W x MeJA, MeJA x ET and W x MeJA x ET treatments. Three genes (*HbERF-IXa1*, *HbERF-IXb1* and *HbERF-IXb2*) were significantly regulated by the MeJA and ET treatment. Although the expression ratio for the *HbERF-IXc4* gene was dramatically increased for the combined MeJA x ET and W x MeJA x ET treatments, only the interaction between W and ET was significant in this analysis, but not for MeJA and ET.

**Fig 7:**
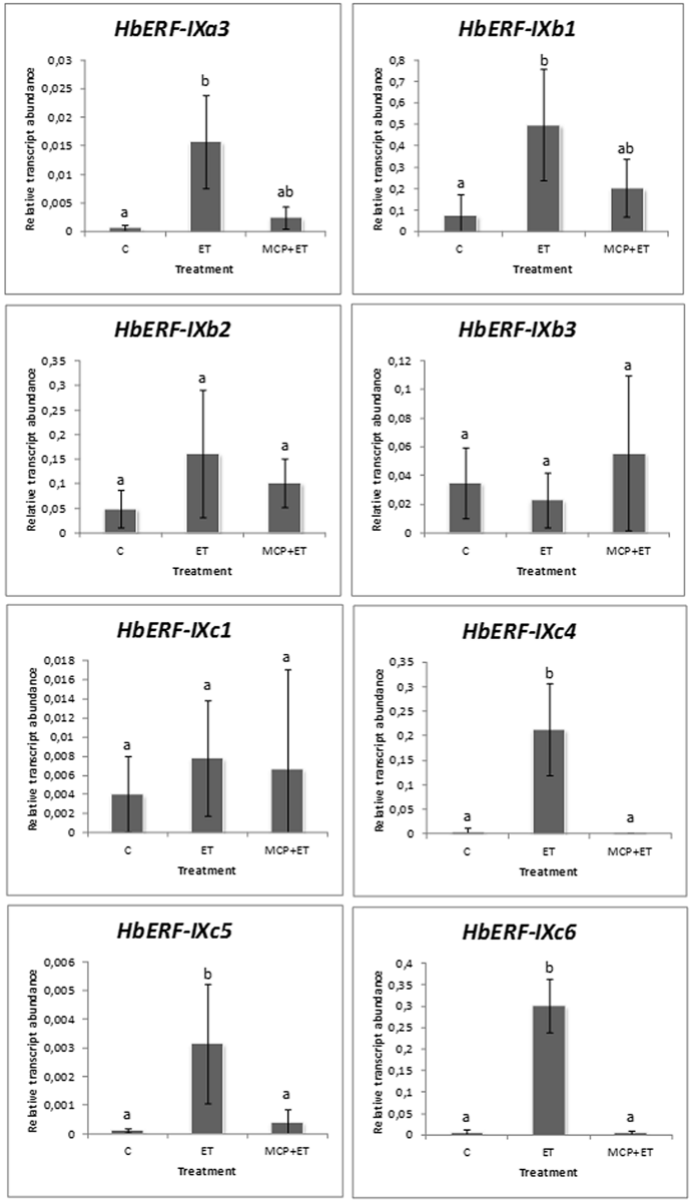
Effect of 1-MCP treatment on the relative transcript abundance of 8 genes of *Hevea brasiliensis* ERF group IX.

The effect of an ethylene action inhibitor, 1-MCP, was tested on the relative transcript abundance of 47 *AP2/ERF* genes in order to check the specificity of the ethylene induction of these genes. The transcript abundance of seven of these genes was reduced or abolished in 1-MCP + ET-treated plants (S8 Table). Three out of 8 genes from ERF group IX (*HbERF-IXc4*, *HbERF-IXc5*, and *HbERF-IXc6*) showed a significant inhibition of ethylene induction by 1-MCP (Fig 7). By contrast, the transcript accumulation of 5 genes of ERF group IX (*HbERF-IXa3*, *HbERF-Xb1*, *HbERF-IXb2*, *HbERF-IXb3*, and *HbERF-IXc1*) was not affected by 1-MCP pre-treatment, revealing an indirect action of ethylene.

## Discussion

### Latex harvesting-induced mechanisms developed for the response to abiotic stress

The technique for latex harvesting in rubber trees is a unique feature that requires recurrent tapping to allow latex flow, and regular applications of ethephon for some *Hevea* clones with a low laticifer metabolism to stimulate latex production. This mechanism is achievable because rubber trees can tolerate repeated tapping [29]. Tapping induces various types of stress, such as mechanical wounding, osmotic stress due to latex flow and consequent laticifer plasmolysis, as well as metabolic activity necessary for latex regeneration between two tappings. With regard to ethephon, the application of exogenous ethylene induces the activation of metabolic defences.

These dramatic harvesting stresses result in complex regulation following responses observed for various types of abiotic stress. As many types of abiotic stress ultimately result in cell desiccation and osmotic imbalance [30], there is an overlap of stress responses. Most likely, similar mechanisms could take place during latex harvesting, which means that the effect of tapping and ethephon stimulation is not specific. In this study, most of the genes induced by tapping were induced by dehydration and only some of them were specifically triggered by wounding, methyl jasmonate or ethylene. Likewise, most of the ethephon-induced ERFs were induced by dehydration resulting in osmotic stress. For example, three genes uniquely regulated by ethephon in latex (*HbERF-IVa3* and *HbERF-VIIIa13*) and in bark (*HbERF-VIIIa10*) were induced by dehydration. This result suggests that harvesting stress generates predominantly osmotic stress, which is translated into several hormonal responses.

Members of several ERF groups could be major actors in the response to harvesting stress. Indeed, twenty genes belonging to ERF groups I, II, IV, VI, VII, VIII, IX and X were differentially regulated during these stresses and considered as expression marker genes. The genes of ERF groups III and IV, which correspond to CBF/DREB according to the Sakuma classification, were regulated under osmotic stress during acclimatization against cold and dehydration [13,31,32]. Of these two groups, only *HbERF-IVa3* was identified as expression marker genes. Induced by cold and dehydration in juvenile plants, *HbERF-IVa3* transcripts were highly accumulated in response to tapping in latex. Two other genes, *HbERF-VIIIa9* and *HbERF-VIIIa10*, also had the same pattern of induction by dehydration and cold. The promoter analysis of these genes has been carried out using genomic sequence from another *Hevea* clone CATAS 7-33-97. Consequently, gene expression analyses for clone PB 260 and prediction from promoter analyses for clone CATAS 7-33-97 must be carefully compared. These genes harboured CRT/DRE and LTRE *cis*-acting elements (data not shown), which are involved in the response to cold, dehydration and low temperature [33,34]. This result suggests a regulation of *HbERF-IVa3*, *HbERF-VIIIa9* and *HbERF-VIIIa10* by osmotic stress in latex after tapping. Transcripts of *HbERF-VII* genes were abundant in latex [18]. HbERF-VIII and HbERF-IX members induced during latex harvesting showed more complex regulation. Most of the *HbERF-VIII* genes were repressor-type transcription factors [35]. The expression marker gene, *HbERF-VIIIa10* was orthologous to *AtERF11*, a negative regulator for ABA-mediated control of ethylene synthesis. Its expression during latex harvesting suggests a certain negative control of the effect of osmotic stress in laticifers. Differential responses of several *HbERF-IX* genes showed possible multilayer regulation (see later in the discussion). For ERF group X, *HbERF-Xb1* had a high transcript accumulation in latex after tapping and ethephon stimulation, and interestingly was found to be orthologous to *RRTF1*, which was demonstrated to be a regulator of redox status [36]. Taken all together, these results suggest that osmotic stress occurring during latex flow and consequent hormone biosynthesis might play an important role in the regulation of *HbERF* genes.

### Putative involvement of hormonal, reactive-oxygen species and nitric oxide signalling pathways in the response to harvesting stress

The response to harvesting stress used potentially hormonal, reactive-oxygen species (ROS) and nitric oxide (NO) signalling pathways. Harvesting stress could induce the biosynthesis of hormones. The biosynthesis of ethylene is induced by wounding [37] but also by osmotic stress and exogenous ethylene from ethephon application [29]. Several ERFs are known to activate the biosynthesis of hormones. Recently, one member of ERF group II, ORA47, was postulated as a positive regulator of JA biosynthesis [38]. The expression of the *ORA47* gene was reported to be induced by JA in a COI1-dependent manner [39]. In *Hevea*, *HbERF-IIb2*, an orthologue to ORA47, was also induced by JA and might have a similar role in laticifers.

ERFs, which were previously described as ethylene-dependent transcription factors, are also shown to be regulated by various hormonal signalling pathways. In *Hevea*, although most of the *ERF* genes were induced by ethylene and/or ethephon, several of them are likely to be ethylene independent. Firstly, *HbERF-Ib7*, *HbERF-VIIIa8*, and *HbERF-VIIIa9* were not induced by either ethephon in mature trees or by ethylene in juvenile plants. Secondly, some other genes (*HbERF-IIb2*, *HbERF-IVa*, *HbERF-VI5*, *HbERF-VIIa20*, *HbERF-VIIIa10*, *HbERF-VIIIb1*, *HbERF-IXa3*, *HbERF-IXb3*, *HbERF-IXc1*, *HbERF-Xa8*, and *HbERF-Xb1*) were induced by both ethylene and other treatments. Given that an ethylene inhibitor could not hamper the expression of these genes, an indirect effect of ethylene, or too low a 1-MCP concentration, were suspected. In addition, contigs related to hormonal biosynthesis genes were found in the *Hevea* clone PB 260 transcriptome database [18], which showed the existence of hormonal biosynthesis pathways in *Hevea* (Table S9 in [18]). Furthermore, *in silico* analysis of promoters of *HbERF* genes confirmed the presence of *cis-*acting regulatory elements related to hormonal signalling. Ethylene-response elements were putatively identified in all members of HbERF group IX suggesting a potential primary response in the ethylene transduction pathway. The presence of 1–2 copies of the GCC-box suggests that *HbERF-IXa3*, *HbERF-IXb1*, *HbERF-IXb2*, *HbERF-IXc4*, and *HbERF-IXc5* could be targeted by other ERFs.

Free radicals such as ROS and NO are considered as secondary messengers that regulate ERFs. Members of ERF-VII (RAP2.3 and RAP2.12) were able to perceive both hypoxia and NO signals and to integrate the N-end rule pathway for protein degradation [40] whereas ERF6 (group ERF-IXb) and RRTF1 (group ERF-Xb) detected ROS or redox status [36,41]. NO is synthesized during tissue damage. In *Hevea*, NO is assumed to be related to the cyanogenesis process [42]. At a certain level, this process could lead to bark necrosis and cessation of latex flow [43]. With a stronger effect of tapping than ethephon, a high transcript accumulation of HbERF-VII after tapping suggests the existence of antioxidant transcriptional regulations in wounded latex cells. The induction of two *ERF* genes from group IX (*ERF1* and *ERF2*) occurred during reoxygenation after hypoxia in *Arabidopsis* [44]. The antioxidant-responsive element 1 is a regulatory motif of redox sensing mechanisms [45,46]. In *Hevea*, the promoter regions of *HbERF-IXc4* and *HbERF-IXc5* have an ARE1 *cis*-acting element revealing their putative involvement in the response to oxidative stress.

### The *ERF* genes from group IX potentially play an important role in laticifers

Among the other ERF groups, many HbERF-IX members were strongly regulated by harvesting and abiotic stress. The presence of various *cis-*acting regulatory elements in *HbERF-IX* promoters suggested an activation of these genes by ethylene, jasmonate, auxin, cytokinin, gibberellins, abscisic acid, and oxidative stress. The high level of expression of *HbERF-IX* genes might be related to the absence of an intron, which is also observed in *ERF-IX* genes from other plant species [16]. The absence of an intron could result in rapid and constitutive gene expression required upon stress [47].

From a previous analysis, the orthologues in *Arabidopsis* of five members of HbERF-IX were predicted: *HbERF-IXa3* with *AtERF1*, *HbERF-IXb3* with *ERF5/MACD1*, *HbERF-IXc4* and *HbERF-IXc5* with *ERF1*, and *HbERF-IXc6* with *ORA59* [18]. The induction of *HbERF* genes by ET and MeJA suggests a role in the crosstalk between these two hormones. For example, the high expression of *Arabidopsis* ERF1 depends on the combination of ET and JA [48]. The two *Hevea* orthologues to ERF1 had a similar expression profile. Both the JA and ET treatments could trigger *HbERF-IXc4* and *HbERF-IXc5* expression and the combination of both JA and ET resulted in a synergetic effect. Although ERF1 is at the crosstalk of the signalling pathways of ET and JA, the expression of its orthologues in *Hevea* was strictly inhibited by an ethylene inhibitor (1-MCP). These genes were demonstrated to be dramatically induced by the combination of ET and JA, suggesting that the jasmonate signalling pathway might intervene upstream of ethylene. The ABA signalling pathway could also be upstream of ET and JA [49]. Intriguingly, this recent study demonstrated that ABA treatment extinguished the combinatory effect of ET and JA on ERF1. Given the two *Hevea* orthologues to ERF1 showed strong expression during dehydration, HbERF-IXc4 and HbERF-IXc5 could be related to the regulation of ABA during osmotic stress. This hypothesis should be followed up by further analysis on ABA interaction with ET and JA.

*HbERF* genes might play a major role in the regulation of latex production. Firstly, ORA59 and ERF1 are known to induce the expression of defence genes, and in particular *PDF1*.*2*, which harbour the GCC-box in their promoter sequences [48,50]. Given *HbERF-IXc4* and *HbERF-IXc5* were able to transactivate the GCC-box, their involvement in the control of defence genes in laticifers is assumed. Secondly, this kind of regulation has also been illustrated in the control of redox status *via* RRTF1. This gene induced a cluster containing GCC-box target-genes during the occurrence of redox imbalance [36]. The *Hevea* orthologue, *HbERF-Xb1*, should play a role in the normal adjustment of redox status in laticifers after latex harvesting. Thirdly, water exchanges between the inner liber and latex cells is promoted by *HbPIP2;1* and *HbTIP1;1* [2]. For example, an ERF called *TRANSLUCENT GREEN* has been shown to regulate aquaporin genes directly [51]. An analogous mechanism should exist for sucrose loading since several sucrose transporters have been demonstrated to be induced by ethylene [52]. Lastly, induction of the biosynthesis pathways of secondary metabolites always requires hormonal interaction between JA and others. Thus, many ERFs are known to be dependent on ethylene and jasmonate. For example, in *Catharanthus roseus* and *Nicotiana tabaccum*, ORCA3 has been shown to be a master regulator of secondary metabolism during jasmonate responses [53]. The involvement of jasmonate in the biosynthesis of terpene, a defence molecule, has been highlighted [54]. As natural rubber biosynthesis follows that route, the genes of *ERF* group IX are assumed to play an important role in regulating latex cell metabolism. Further functional analysis should lead to the identification of target genes related to latex production and tapping panel dryness.

## Supporting Information

S1 Table List of primer sequences for 47 genes of the *Hevea brasiliensis* AP2/ERF superfamily. (XLSX)

S2 Table Comparison of Cp values, standard deviation and coefficient of variance for gene expression analysis by real-time RT-PCR of 11 housekeeping genes in 30 different samples of matures trees and juvenile plants. (XLSX)

S3 Table Relative transcript abundance profile of 47 genes of the *Hevea brasiliensis* AP2/ERF superfamily in latex and bark tissues of mature trees during latex harvesting. The relative transcript abundance was measured by real-time RT-PCR. Tapping without ethephon (Eth-) and with ethephon (Eth +) were applied to both studied tissues. Values are the means of three biological replicates. Values of relative transcript abundances were analysed with XLSTAT after LOG(X) transformation. The statistical analysis was performed with an ANOVA followed by the Student Newman-Keuls test.
(XLSX)

S4 Table Interaction analysis profile of 47 genes of the *Hevea brasiliensis* AP2/ERF superfamily in latex and bark tissues of mature trees during latex harvesting. Statistical analysis of the tissue effect was performed using an ANOVA followed by a Newman-Keuls test. The level of interaction between treatments was assessed for each tested gene. The data correspond to F values. P-values for significant data are indicated using asterisks as follows: 0.001 (***); 0.01 (**); 0.05 (*); and 0.1 (°). T: tapping; Eth: Ethephon TxEth: combination of both.
(XLSX)

S5 Table Genomic scaffold analysis for 35 genes for *Hevea* ERFs. Contig sequences were generated from 454 RNA sequencing (Duan et al, 2013; Piyatrakul et al, submitted). Genomic scaffolds from clone CATAS 7-33-97 were provided by the Beijing Institute of Genomics.
(XLSX)

S6 Table Number of copies of *cis*-acting regulatory elements involved in the HbERF-IX promoter region using an *in silico* pattern matching search against PLACE. The analysis was carried out in the -2kb region upstream of the start codon (ATG).
(XLSX)

S7 Table Summary of the analysis of variance tables for each tested gene from *Hevea brasiliensis* ERF group IX in 3-month-old epicormic shoots of clone PB 260. The data correspond to F values. P-values are indicated as follows: 0.001 (***); 0.01 (**); 0.05 (*); 0.1 (°).
(XLSX)

S8 Table Effect of 1-MCP treatment on the relative transcript abundance of 47 genes of the *Hevea brasiliensis* AP2/ERF superfamily. The values of the relative transcript abundance were analysed with XLSTAT software after LOG(X) transformation. The statistical analysis was performed with an ANOVA followed by the Student Newman—Keuls test. ND refers to non-determined data.
(XLSX)
